# CD19 exon 2 skipping is a potential prognostic correlate of anti-CD19 CAR-T therapy relapse

**DOI:** 10.3389/fmmed.2026.1763390

**Published:** 2026-02-12

**Authors:** Søren Helweg Dam, Giorgia Moranzoni, Magnus Haraldson Høie, Signe Modvig, Karin A. W. Wadt, Bodil Als-Nielsen, Kjeld Schmiegelow, Kristoffer Vitting-Seerup, Mike Bogetofte Barnkob, Lars Rønn Olsen

**Affiliations:** 1 LEO foundation Skin Immunology Research Center, Department of Immunology and Microbiology, University of Copenhagen, Copenhagen, Denmark; 2 Department of Health Technology, Technical University of Denmark, Kongens Lyngby, Denmark; 3 Department of Clinical Immunology, Copenhagen University Hospital Rigshospitalet, Copenhagen, Denmark; 4 Department of Clinical Medicine, Copenhagen University, Copenhagen, Denmark; 5 Department of Clinical Genetics, Copenhagen University Hospital Rigshospitalet, Copenhagen, Denmark; 6 Department of Pediatric and Adolescent Medicine, Copenhagen University Hospital Rigshospitalet, Copenhagen, Denmark; 7 Centre for Cellular Immunotherapy of Haematological Cancer Odense (CITCO), Department of Clinical Immunology, Odense University Hospital, University of Southern Denmark, Odense, Denmark

**Keywords:** CAR T-cell therapy, CD19, isoform switching, relapse prediction, splice variant

## Abstract

Relapse following anti-CD19 chimeric antigen receptor (CAR) T cell therapy remains a concern in the treatment of refractory B-cell malignancies. Although the CD19**Δ**exon2 splice variant has been linked to treatment failure, reliable pre-treatment biomarkers for relapse risk are lacking. Here, we analyzed RNA-sequencing data from a small publicly available cohort of four anti-CD19 CAR-T-treated B-cell acute lymphoblastic leukemia patients, including one responder, one non-responder, and two who relapsed after initial response. We quantified the percent spliced in (PSI) of CD19 exon 2, as a proxy for CD19**Δ**exon2 abundance before and after treatment. The patient with the lowest pre-treatment exon 2 PSI (i.e., highest estimated abundance of CD19**Δ**exon2) experienced the earliest relapse, whereas the complete responder showed no detectable exon 2 skipping. In silico protein structure modeling indicated reduced structural stability of the FMC63 epitope region in the CD19**Δ**exon2 variant, supporting a potential mechanistic link between exon 2 exclusion and antigen escape. Analysis of larger RNA-sequencing datasets from CAR-T treatment-naïve B-cell malignancies and healthy tissues revealed low-level exon 2 skipping in some individuals across both malignant and normal B cells. These findings suggest that CD19 exon 2 skipping may correlate with relapse after CAR-T therapy, and its presence in treatment-naïve individuals highlights its potential for evaluation as an RNA- or qPCR-based biomarker in future studies.

## 1 Introduction

Chimeric antigen receptor (CAR) T cell therapy has reshaped treatment options for refractory B-cell malignancies (Cao et al., 2018; Di Blasi et al., 2022; Frontzek et al., 2022; Gardner et al., 2017; Lee et al., 2015; Li et al., 20118b; Majzner and Mackall, 2019; Maude et al., 2014; Maude et al., 2018; Park et al., 2018; Schuster et al., 2019; Turtle et al., 2016; Xu et al., 2019; Zhang et al., 2019; Zhang et al., 2020). These therapies use autologous T cells engineered to express a CAR that targets the B-cell surface protein CD19 (Frontzek et al., 2022; Grupp et al., 2013; Schuster et al., 2019; Zhang et al., 2020). CD19 CAR-T products have achieved high complete remission rates in B-cell acute lymphoblastic leukemia (B-ALL; ∼70–90%) (Davila et al., 2014; Lee et al., 2015; Maude et al., 2014; Nguyen et al., 2008; Park et al., 2018; Roddie et al., 2024; Turtle et al., 2016; Zhang et al., 2020) and diffuse large B-cell lymphoma (DLBCL; ∼50–75%) (Abramson et al., 2020; Bender et al., 2024; Bouchkouj et al., 2022; Frontzek et al., 2022; Locke et al., 2020; Locke et al., 2019). Nevertheless, a substantial proportion of patients eventually relapse after CD19 CAR-T therapy (B-ALL: ∼40–50% (Bonifant et al., 2016; Davila et al., 2014; Frontzek et al., 2022; Gauthier and Turtle, 2018; Grupp et al., 2013; Maude et al., 2018; Schuster et al., 2019; Turtle et al., 2016; Xu et al., 2019; Yang et al., 2018; Zhang et al., 2019); DLBCL: ≤66% (Abramson et al., 2020; Bethge et al., 2022; Di Blasi et al., 2022; Iacoboni et al., 2021; Locke et al., 2019; Nastoupil et al., 2020; Neelapu et al., 2017; Pasquini et al., 2020; Schuster et al., 2019)).

The mechanisms underlying relapse are multifactorial and incompletely understood (Xu et al., 2019). While limited CAR-T cell persistence can contribute to treatment failure (Johnson and Locke, 2023; Nie et al., 2020), several studies have identified loss or alteration of the CD19 antigen as a major cause of resistance (Asnani et al., 2020; Bagashev et al., 2018; Braig et al., 2017; Cortés-López et al., 2022; Fischer et al., 2017; Hamieh et al., 2019; Jacoby et al., 2016; Ledererova et al., 2021; Orlando et al., 2018; Rabilloud et al., 2021; Sotillo et al., 2015; Zhao et al., 2021). All currently approved CD19 CAR-T therapies employ an FMC63-derived single-chain variable fragment that recognizes an epitope encoded by CD19 exons 3 and 4 (Grupp et al., 2013; He et al., 2023; Klesmith et al., 2019; Nicholson et al., 1997; Roddie et al., 2024; Zola et al., 1991). Alternative splicing events in CD19 can destabilize the structure of this region and impair CAR binding (Asnani et al., 2020; Bagashev et al., 2018; Cortés-López et al., 2022; Sotillo et al., 2015). Among these, the CD19**Δ**exon2 variant has been linked to therapy resistance and relapse following CD19 CAR-T treatment. Despite this established association, patients are not currently stratified before therapy based on CD19 splicing patterns, and no validated pre-treatment biomarkers exist to predict relapse risk.

In this study, we investigated whether CD19 exon 2 skipping detectable at the mRNA level could serve as a correlate of CD19 CAR-T therapy outcomes. Specifically, we quantified the percent spliced in (PSI) of CD19 exon 2, calculated from exon 1–3, exon 1–2, and exon 2–3 splice-junction reads, in a small publicly available cohort of B-ALL patients treated with CD19 CAR-T therapy. We further examined the structural implications of exon 2 skipping using *in silico* protein modeling and assessed its baseline prevalence in B-ALL, DLBCL, and healthy blood and spleen samples. This approach aims to determine whether pre-treatment splicing patterns can inform relapse risk and whether qPCR-based detection of exon 2 skipping could provide a practical biomarker for clinical monitoring.

## 2 Materials and methods

### 2.1 Datasets

Five datasets were analyzed in this study (Table 1). Raw and processed RNA-seq data were obtained from publicly available repositories or existing institutional cohorts.

**TABLE 1:** Overview of datasets analyzed in this study.

Dataset	Samples (n)	Data type	Median age (range)	% Male	Source
B-ALL	75	Raw reads	3 (0–17)	60.3%	iCOPE/STAGING
DLBCL	553	Snapcount	63 (18–86)	52.2%	GOYA (McCord et al., 2019; Vitolo et al., 2017)
CD19 CAR-T–treated B-ALL	4	Raw reads	52 (29–59)	50%	Zhang et al. (Zhang et al., 2019)
Healthy blood	1,048	Snapcount	53 (20–79)	65.8%	GTEx v2 (Lonsdale et al., 2013)
Healthy spleen	255	Snapcount	52 (20–79)	63.9%	GTEx v2 (Lonsdale et al., 2013)

#### 2.1.1 Treatment-naïve B-cell acute lymphoblastic leukemia (B-ALL) cohort

The B-ALL cohort comprised 75 pre-treatment bone marrow samples collected as part of the iCOPE/STAGING study (Capital Region Ethics Committee ID H-15016782). Patients were diagnosed with B-ALL prior to therapy initiation. RNA was extracted and sequenced on an Illumina platform using 100-bp paired-end reads. Raw FASTQ files were downloaded from the European Genome-phenome Archive and processed as described in RNA sequencing and splicing quantification. This cohort was used to establish the baseline distribution of exon 2 inclusion in treatment-naïve B-ALL.

#### 2.1.2 CAR-T–treated B-ALL cohort

A small publicly available dataset of four B-ALL patients treated with CD19 CAR-T therapy was analyzed to assess exon 2 skipping in the therapeutic context. For each patient, RNA-seq was performed on pre- and post-treatment bone marrow aspirates (Zhang et al., 2019). Raw FASTQ files were retrieved from Genome Sequence Archive (accession CRA000746) (Wang et al., 2017) and processed as described below. Patient characteristics are summarized in Table 2, including response category, relapse time, and tumor burden (TB) at day 0. The cohort included one complete responder (CR), one non-responder (NR), and two patients who relapsed after an initial response (R1, R2). All four CAR-T–treated B-ALL patients from the Zhang cohort had relapsed or refractory disease and had received multiple prior lines of therapy. Immediately before CAR-T infusion, all patients underwent lymphodepleting conditioning with fludarabine (30 mg/m^2^) and cyclophosphamide (750 mg/m^2^) for 5 consecutive days, as described in the original study. This pre-treatment regimen, together with substantial variation in baseline bone marrow blast fractions (0.25%–65.46%), resulted in marked heterogeneity in CD19 expression and B-cell representation in the RNA-seq data. These clinical and biological factors were therefore expected to influence CD19 read depth and junction coverage, and were taken into account when interpreting exon-skipping metrics in this cohort. Sashimi plots were generated to visualize exon 1–4 splice junction coverage. Due to the low CD19 read depth in two of the samples, no minimum-junction-depth filter was applied to this cohort. Exon 1–3 junction counts were therefore reported descriptively as indicators of exon 2 skipping. PSI values were not calculated for samples with extremely low CD19 coverage, as the denominator of the PSI formula becomes unstable under near-zero read depth and would not reliably reflect exon usage in these specimens.

**TABLE 2:** Clinical characteristics and treatment response of CD19 CAR-T–treated B-ALL patients included in this study. Tumor burden (TB) indicates the proportion of leukemic blasts in bone marrow at the time of sampling, as reported in the original study.

Patient	Responder	Relapse	TB (%) day 0
R1	Yes	1.5 months	17.92
R2	Yes	13 months	0.25
NR	No	N/A	65.46
CR	Yes	N/A	0.5

#### 2.1.3 Diffuse large B-cell lymphoma (DLBCL) cohort

RNA-seq data for 553 DLBCL cases were obtained via snapcount (v1.14.0) (Charles, 2023) from the GOYA clinical trial dataset (McCord et al., 2019; Vitolo et al., 2017) available in the SRA database (Leinonen et al., 2011) (SRP183071). The GOYA cohort includes newly diagnosed, treatment-naïve DLBCL patients with paired genomic and transcriptomic profiling. The samples were classified as activated B-cells (n = 151), germinal center B-cells (n = 298), or unclassified (n = 104), as described by McCord et al. (2019). Junction-level quantifications were retrieved from the recount3 data repository using snapcount’s API interface, and CD19 exon-junction reads were extracted for PSI estimation. These data provided an independent lymphoma cohort for comparison with B-ALL and healthy B-cell–rich tissues.

#### 2.1.4 Healthy tissue cohorts

Healthy whole blood (n = 1,048) and spleen (n = 255) RNA-seq samples were obtained from GTEx v8 (Lonsdale et al., 2013). Both these tissues were selected because they contain abundant mature B-cell populations, enabling comparison of exon 2 inclusion under normal physiological conditions. Splice junction counts for CD19 exons 1–2, 1–3, and 2–3 were retrieved through snapcount (v1.14.0) (Charles, 2023) and used to compute PSI values following the same workflow applied to malignant datasets.

### 2.2 RNA sequencing and splicing quantification

For DLBCL and healthy samples, splice junction counts were obtained from snapcount (v.1.14.0) (Charles, 2023), which compiles exon–exon junction quantifications from large public RNA-seq collections (GOYA, GTEx). For the two B-ALL cohorts, reads were trimmed using fastp (v.0.21.1) (Chen et al., 2018) and aligned to the human genome (GRCh38, release 107) with STAR (v.2.7.10a) (Dobin et al., 2013), and outrigger (v.1.1.1) (Song et al., 2017) was used to compute CD19**Δ**exon2 PSI values.

Splice junction reads corresponding to CD19 exons 1–2, 1–3, and 2–3 were extracted from aligned BAM files. The PSI value for exon 2 was calculated as:

$$PSI\;exon\;2=\frac{\left(junction\_reads\_\left\{exon1-2\right\}+junction\_reads\_\left\{exon2-3\right\}\right)}{\left(junction\_reads\_\left\{exon1-2\right\}+junction\_reads\_\left\{exon1-3\right\}+junction\_reads\_\left\{exon2-3\right\}\right)}$$

where a lower PSI reflects increased exon 2 skipping. In the treatment-naïve B-ALL, DLBCL, blood, and spleen cohorts, PSI was calculated only for samples with ≥30 splice-junction reads in the CD19 exon 1, 2, and 3 region, ensuring robust estimation of exon inclusion.

### 2.3 Structural modeling

The protein structure of full-length CD19 was retrieved from the AlphaFold Database (AlphaFold accession AF-P15391-F1-v6), while the CD19**Δ**exon2 variant structure was predicted *de novo* using AlphaFold3 via template-free modeling. (Abramson et al., 2024). The protein sequences were aligned with the R package msa (v.1.36) (Bodenhofer et al., 2015) and predicted the topology of each isoform using DeepTMHMM (v.1.0) (Hallgren et al., 2022). Per-residue model confidence was assessed using pLDDT scores provided by AlphaFold. These scores serve as estimates of local model confidence rather than direct measures of biophysical stability (Binder et al., 2022).

### 2.4 Visualization and statistical analysis

All computational analyses and figure generation were performed in R (v4.3.1) and Python (v3.10). Sashimi plots were generated using ggsashimi (v.1.1.5) (Garrido-Martín et al., 2018), and histograms of PSI distributions were plotted with ggplot2. No formal hypothesis testing was applied due to limited sample size in the CAR-T–treated cohort; all findings were interpreted descriptively.

## 3 Results

### 3.1 CD19**Δ**exon2 disrupts the spatial continuity of the FMC63 epitope compared with canonical CD19

**FIGURE 1:**
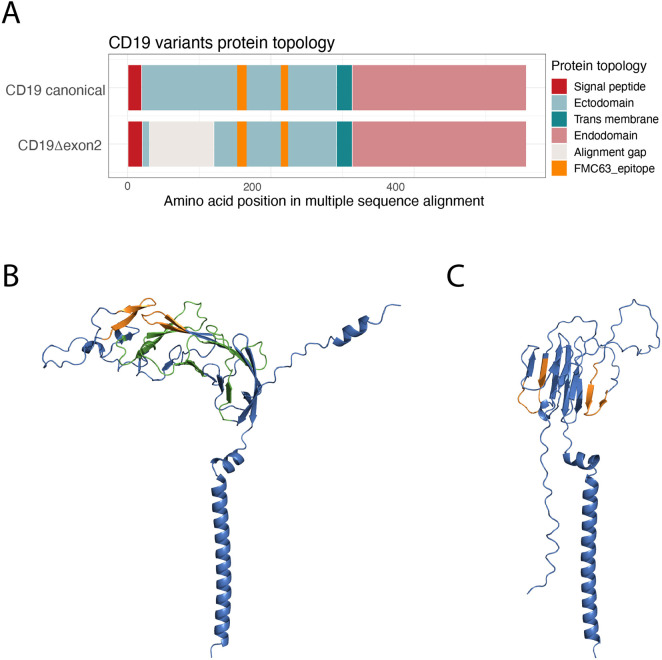
**(A)** Schematic alignment of the membrane topology of canonical CD19 and CD19**Δ**exon2, predicted using DeepTMHMM. The discontinuous FMC63 epitope is indicated in orange. **(B)** Experimentally resolved 3D structure of canonical CD19 and **(C)** AlphaFold-predicted structure of CD19**Δ**exon2, generated using template-free modeling. The FMC63 epitope (orange) and exon 2 (green) are highlighted. In the canonical structure, the two epitope segments fold into close spatial proximity, forming a continuous binding surface for FMC63. In contrast, in the CD19**Δ**exon2 model this spatial arrangement is disrupted, providing a plausible structural explanation for the reduced efficacy of FMC63-based CAR-T therapy. The endodomain of both variants is omitted for clarity.

To assess the structural consequences of exon 2 skipping, we first compared the domain topology of canonical CD19 and the predicted Δexon2 variant (Figure 1A). The alignment shows that removal of exon 2 shortens the extracellular portion of CD19 while leaving the FMC63-recognized epitope, encoded by exons 3–4, intact. Structural modeling combining the experimentally resolved structure of canonical CD19 with an AlphaFold-predicted model of CD19Δexon2 revealed clear geometric differences within the FMC63 epitope region (Klesmith et al., 2019) (Figures 1B,C, the pLDDT of the structures is shown in Supplementary Figure S1). In the canonical structure, the two discontinuous epitope segments fold into close spatial proximity, forming a contiguous binding surface for FMC63. In contrast, exon 2 skipping disrupts this spatial arrangement, resulting in a greater separation of the epitope elements and suggesting impaired antibody accessibility. This structural alteration provides a plausible mechanistic explanation for the experimentally observed reduction in FMC63 binding affinity to CD19Δexon2, supporting exon 2 as a stabilizing element of the functional epitope. Having established that exon 2 skipping likely negatively affects the structural integrity of the FMC63 epitope, we next examined whether this event could be detected in pre-treatment RNA samples from CD19 CAR-T–treated patients.

### 3.2 CD19 exon 2 skipping correlates with relapse in a small cohort of CD19 CAR-T treated patients

**FIGURE 2:**
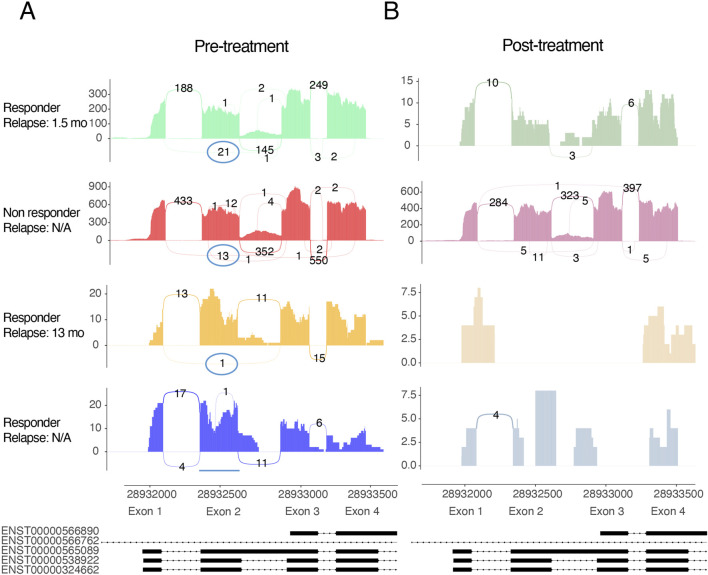
Sashimi plots of CD19 exon 1–4 splice junctions in four CD19 CAR-T–treated B-ALL patients. **(A)** Pre-treatment splice-junction profiles for the rapid-relapse patient, non-responder, slow-relapse patient, and complete responder. The exon 1–3 junction, indicating exon 2 skipping, is prominently detected in the rapid-relapse patient, present at intermediate levels in the non-responder, minimal in the slow-relapse patient, and absent in the complete responder. **(B)** Post-treatment splice-junction profiles. CD19 read coverage is markedly reduced in all patients who initially responded to therapy, consistent with B-cell depletion, whereas the non-responder retains comparatively higher coverage. Purple circles highlight exon 1–3 junctions corresponding to the CD19**Δ**exon2 splice event.

To explore whether exon 2 skipping occurs in CD19 CAR-T treated patients and whether it may quantitatively relate to treatment outcome, we analyzed RNA-sequencing data from a small publicly available cohort of four B-ALL patients representing one complete responder, one non-responder, and two who relapsed after initial remission (Table 2) (Zhang et al., 2019). For each sample, we quantified exon 1–3 junction reads as a proxy for exon 2 skipping, since the junction read coverage in these is too low to provide stable PSI calculations. Before treatment, sashimi plots revealed substantial exon 1–3 coverage in the rapid-relapse patient (21 reads) and detectable usage in the non-responder (13 reads), whereas the slow-relapse patient showed only minimal exon 2 skipping (1 read) and the complete responder showed none (0 reads) (Figure 2A). Post-treatment samples exhibited markedly reduced CD19 read coverage in all patients who demonstrated an initial response to therapy (rapid relapse, slow relapse, complete responder), consistent with effective B-cell depletion following CAR-T infusion. In contrast, CD19 coverage declined only modestly in the non-responder, who also retained exon 1–3 junction usage (Figure 2B). Pre-treatment PSI values reflected the same ordering, with the rapid-relapse patient showing the lowest exon 2 inclusion and the complete responder showing no evidence of skipping. Although the small cohort precludes statistical inference, the qualitative concordance between exon 2 inclusion and clinical outcome suggests that reduced exon 2 retention may be associated with CD19 CAR-T therapy failure. To determine whether exon 2 skipping occurs only in the context of CAR-T therapy or is also present at baseline, we next examined its prevalence in larger cohorts of treatment-naïve malignant and healthy B-cell–rich tissues.

### 3.3 CD19**Δ**exon2 is detectable at low levels in treatment-naïve B-cell malignancies and normal B-cell rich tissues

**FIGURE 3:**
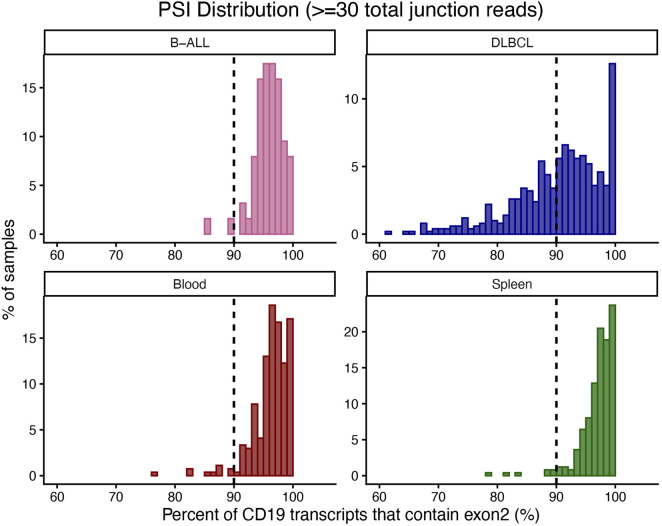
Distribution of CD19 exon 2 inclusion across treatment-naïve malignant and healthy B-cell–rich tissues. Histograms showing the percent spliced in (PSI) of CD19 exon 2 in treatment-naïve B-ALL, DLBCL, healthy peripheral blood, and healthy spleen. Median PSI values were 96.3% (IQR: 94.9–98.6) in B-ALL, 96.9% (IQR: 95.2–98.4) in blood, and 97.7% (IQR: 96.1–98.9) in spleen, indicating consistently high exon 2 inclusion across these tissues. Using a ≥90% PSI threshold to denote near-complete exon retention, 97.3%, 96.3%, and 97.2% of samples in B-ALL, blood, and spleen, respectively, met this criterion. DLBCL displayed substantially greater heterogeneity, with a median PSI of 91.6% (IQR: 86–96); only 59.5% of samples exhibited ≥90% inclusion, while 86.8% showed detectable exon 2 skipping, including a subset with PSI values as low as ∼22%. The broader distribution in DLBCL highlights increased variability in CD19 splicing relative to B-ALL and healthy B-cell populations. The dashed line indicates PSI = 90%. Reported percentages represent the proportion of samples with PSI below this threshold, derived from the histogram distributions.

To evaluate the prevalence of pre-treatment CD19**Δ**exon2 in a broader context, we examined B-cell–rich tissues and malignancies using independent RNA-seq datasets (Figures 3A–D). Here, we quantified the PSI of CD19 exon 2 – a metric that normalizes the exon 1-3 junction reads to exon 1-2 and exon 2-3 junction read depth. CD19 exon 2 inclusion was uniformly high in B-ALL and healthy blood and spleen, with median PSI values of 96.3% (IQR: 94.9–98.6) in B-ALL, 96.9% (IQR: 95.2–98.4) in blood, and 97.7% (IQR: 96.1–98.9) in spleen. Using a conservative threshold of ≥90% inclusion to represent near-complete exon retention until a clinically validated threshold can be defined, 97.3% of B-ALL samples, 96.3% of blood samples, and 97.2% of spleen samples met this criterion. In contrast, DLBCL displayed marked heterogeneity, with a median PSI of 91.6% (IQR: 86–96); only 59.5% of samples exhibited ≥90% inclusion, while 86.8% showed measurable exon 2 skipping, including a DLBCL patient with a PSI value of 22.8%. These results indicate that exon 2 skipping is a rare event in treatment-naïve B-ALL and healthy B-cell–rich tissues but is more frequent and quantitatively variable in DLBCL.

Within each cohort, we tested whether any sample-level covariates (where available: age, sex, prior therapy, and DLBCL subtype) correlate with exon 2 PSI, but found no significant correlations. The presence of measurable CD19**Δ**exon2 transcripts across treatment-naïve malignant and healthy B-cell samples demonstrates that this splicing event occurs at low but detectable levels under physiological conditions. This finding indicates that exon 2 PSI can be robustly quantified from standard RNA-seq data, providing the technical basis for evaluating CD19**Δ**exon2 as a potential pre-treatment biomarker of CAR-T therapy response. Moreover, the detection of CD19**Δ**exon2 in untreated samples supports a model in which this isoform represents a naturally occurring, low-frequency splice variant rather than a strictly therapy-induced transcript (Orlando et al., 2018). Under the selective pressure of CD19-directed CAR-T therapy, malignant cells expressing this pre-existing variant may gain a survival advantage, resulting in apparent enrichment of the isoform in relapsed disease.

## 4 Discussion

Our results support and extend previous studies linking CD19 alternative splicing to CAR-T therapy resistance (Cortés-López et al., 2022; Sotillo et al., 2015). Earlier work has predominantly focused on the post-treatment emergence of the CD19**Δ**exon2 splice variant as a mechanism of antigen escape in B-ALL, often describing it as a resistance-acquired event (Sotillo et al., 2015). However, low-level exon 2 skipping has been noted in some pre-treatment samples and in normal B cells (Fischer et al., 2017), though it has not been systematically quantified or contextualized in larger datasets. Our analysis demonstrates that CD19**Δ**exon2 is consistently detectable at low but measurable levels across treatment-naïve B-ALL and DLBCL samples as well as in healthy B-cell–rich tissues, indicating that this splicing event is a physiologically tolerated feature of CD19 transcription, rather than a disease-specific transcriptional alteration. This observation suggests that the apparent emergence of CD19**Δ**exon2 after therapy may reflect the selective expansion of pre-existing subclones under CAR-T–mediated immune pressure rather than *de novo* induction of the splice variant. Accordingly, CD19 exon 2 skipping is not proposed as a diagnostic or mechanistic biomarker, but a potential correlate of CD19**Δ**exon2 mediated relapse risk. The greater heterogeneity observed in DLBCL compared with B-ALL or healthy tissues may have direct translational relevance as CD19 CAR-T therapy continues to expand into lymphoma indications.

The detection of CD19 exon 2 skipping in treatment-naïve B-cell malignancies as well as in healthy blood and spleen indicates that this splicing event reflects baseline B-cell biology in both homeostasis and disease, rather than a disease-specific transcriptional alteration. Accordingly, CD19 exon 2 skipping is not proposed as a diagnostic biomarker or a general indicator of disease presence. Instead, we propose it as a context-dependent transcript-level correlate that may become clinically relevant under the selective pressure of FMC63-based anti-CD19 therapies, where even low-level expression of CD19Δexon2 could influence treatment response.

Blinatumomab, a bispecific T-cell engager targeting CD19 and CD3, has recently moved toward the forefront of therapy for both pediatric and adult B-ALL (Litzow et al., 2024; Winters and Gore, 2019). As anti-CD19 agents become increasingly integrated into early-line treatment, the need to identify patients at elevated risk of CD19-mediated treatment failure becomes more pressing. Notably, the parental antibody of Blinatumomab, HD37, has been shown to compete with FMC63 for CD19 binding, suggesting a shared or overlapping epitope (Cordoba et al., 2021). This epitope relationship suggests that structural alterations affecting FMC63 recognition, such as those caused by exon 2 skipping, may likewise reduce HD37 engagement. If CD19**Δ**exon2 disrupts the three-dimensional continuity of the HD37 and FMC63 epitopes, as suggested by our structural modeling, then the splice variant may also influence Blinatumomab-mediated cytotoxicity. Although formal testing is required, these observations indicate that exon-level CD19 variation could have broader implications across multiple anti-CD19 therapeutic platforms.

Experimental studies have shown that CD19 isoforms lacking exon 2 exhibit defective folding, abnormal glycosylation, and impaired interaction with the CD81 chaperone complex, resulting in predominant retention within the endoplasmic reticulum rather than efficient trafficking to the plasma membrane (Bagashev et al., 2018). It is likely that only a small fraction of CD19**Δ**exon2 escape ER quality control and reach the cell surface, but this population appears to be unstable and not recognized by CD19-directed antibodies. These observations indicate that exon 2 contributes to proper ectodomain folding and maturation of CD19, consistent with structural modeling, which suggests that exon 2 skipping disrupts the spatial organization and stability of the FMC63 epitope. Together, these findings support a model in which reduced CAR recognition of CD19**Δ**exon2 arises from both impaired trafficking and altered surface conformation, leading to markedly diminished antigen density and lower effective binding affinity.

Several study limitations should be noted. First and foremost, the small number of CAR-T–treated patient samples precludes statistical analysis and prevents definitive conclusions regarding causality or predictive value. Read coverage in post-treatment samples was sparse, reflecting B-cell depletion, which limits sensitive quantification of splicing dynamics with treatment.

A further consideration is the potential confounding effect of pre-treatment tumor burden. High leukemic blast fractions reduce the contribution of normal B cells to total CD19 mRNA, whereas samples with low blast percentages may contain substantial numbers of residual healthy B cells. Because normal B cells express predominantly canonical CD19, variation in the proportion of malignant versus non-malignant B cells will influence the measured exon 2 PSI. In patients with low tumor burden, the presence of many normal B cells could artificially elevate exon 2 inclusion and obscure the true splicing pattern of the leukemic clone. Conversely, in samples dominated by blasts, PSI values may more accurately reflect tumor-intrinsic splicing. These compositional differences complicate direct comparisons across patients and underscore the need for future studies to incorporate parallel measures of tumor purity or single-cell isoform quantification to disentangle malignant and non-malignant contributions to CD19 splicing.

All analyses were performed at the mRNA level, and the protein-level consequences of exon 2 exclusion therefore remain to be verified. Confirmation using FMC63 immunostaining or quantitative proteomic approaches will be essential to determine the exact degree to which reduced exon 2 inclusion translates into decreased surface presentation or altered antibody binding of CD19. An additional limitation of bulk RNA-seq data is that it cannot resolve whether CD19**Δ**exon2 transcripts are uniformly co-expressed with canonical CD19 within the same cells or confined to distinct subclones. If exon 2 skipping occurs stochastically across all B cells, selective pressure from CAR-T therapy would likely have limited impact on overall CD19 expression. However, if a subpopulation preferentially or exclusively expresses CD19**Δ**exon2, such cells could evade recognition and expand under therapeutic pressure. Current single-cell and long-read sequencing technologies are beginning to address this question, but their sensitivity is insufficient to reliably detect the low-abundance exon 1–3 splice junction in complex tissues.

Moreover, while exon 2 usage was quantified using PSI, which provides within-gene normalization and reduces sensitivity to sequencing depth and absolute CD19 expression, we acknowledge that biological covariates such as age, sex, ethnicity, and technical factors including RNA integrity and sample handling could not be systematically assessed across all public datasets due to incomplete metadata.

The structural analyses rely on computational modeling and confidence scoring, which, while informative, require experimental validation to confirm conformational effects on the FMC63 epitope. Future studies incorporating targeted long-read sequencing, isoform-specific qPCR, or biophysical characterization of CD19 variants in larger CD19 CAR-T cohorts prior to treatment could validate these observations and distinguish between stable isoforms and transient splicing intermediates. Despite these constraints, the consistency between structural predictions, patient-level RNA patterns, and baseline tissue distributions strengthens the biological plausibility of the findings. Other CD19 splicing events, including intron 2 retention and alternative exon usage, have also been associated with reduced CAR-T recognition in previous studies. Although our analysis focused on exon 2 skipping, future work should evaluate whether multiple CD19 splice variants act in concert to modulate antigen presentation and therapy response.

From a translational perspective, the detection of exon 2 skipping at the mRNA level offers a feasible route for developing a prognostic biomarker. Since bulk RNA sequencing of leukemic cells contributes to accurate subtyping of B-ALL, and is therefore increasingly being performed as part of the diagnostic workup (Li et al., 2018a), exon 2 PSI could be computationally inferred as an additional feature without extra experimental cost. Alternatively, qPCR assays targeting exon–exon junctions could provide a rapid and inexpensive diagnostic test to stratify relapse risk or monitor splicing changes during follow-up. Larger, prospective studies will be required to establish whether CD19 exon 2 skipping is merely a correlate of poor response or a functional determinant of resistance. Either outcome would enhance understanding of CD19 biology and contribute to optimizing CAR-T patient selection and monitoring. As combinatorial CAR strategies targeting CD19 together with CD20 or CD22 enter clinical testing, understanding the baseline splicing diversity of CD19 may help identify patients who could benefit most from multi-target approaches.

## Data Availability

Publicly available datasets were analyzed in this study. This data can be found here: Data from snapcount compilations are available through the snapcount R package, as used in this study. Data from the CAR-T–treated B-ALL cohort is available from Zhang et al. (2019). Access to the data from the treatment-naïve B-ALL cohort can be requested via the European Genome-phenome Archive (EGAD50000001645).

## Supplementary material

SUPPLEMENTARY FIGURE S1
**(A)** Experimentally resolved 3D structure of canonical CD19 and **(B)** AlphaFold3‐predicted structure of CD19Δexon2, generated using template‐free modeling. Structures are colored by confidence scores (pLDDT): dark blue (>90, high), light blue (70‐90), yellow (50‐70), orange (<50, low). Endodomains are omitted for clarity.
