# Supplementary figures and images for: CD19 exon 2 skipping is a potential prognostic correlate of anti-CD19 CAR-T therapy relapse
Source: Front Mol Med. 2026 Feb 12;6:1763390. doi: 10.3389/fmmed.2026.1763390 (PMC12935319; doi:10.3389/fmmed.2026.1763390)

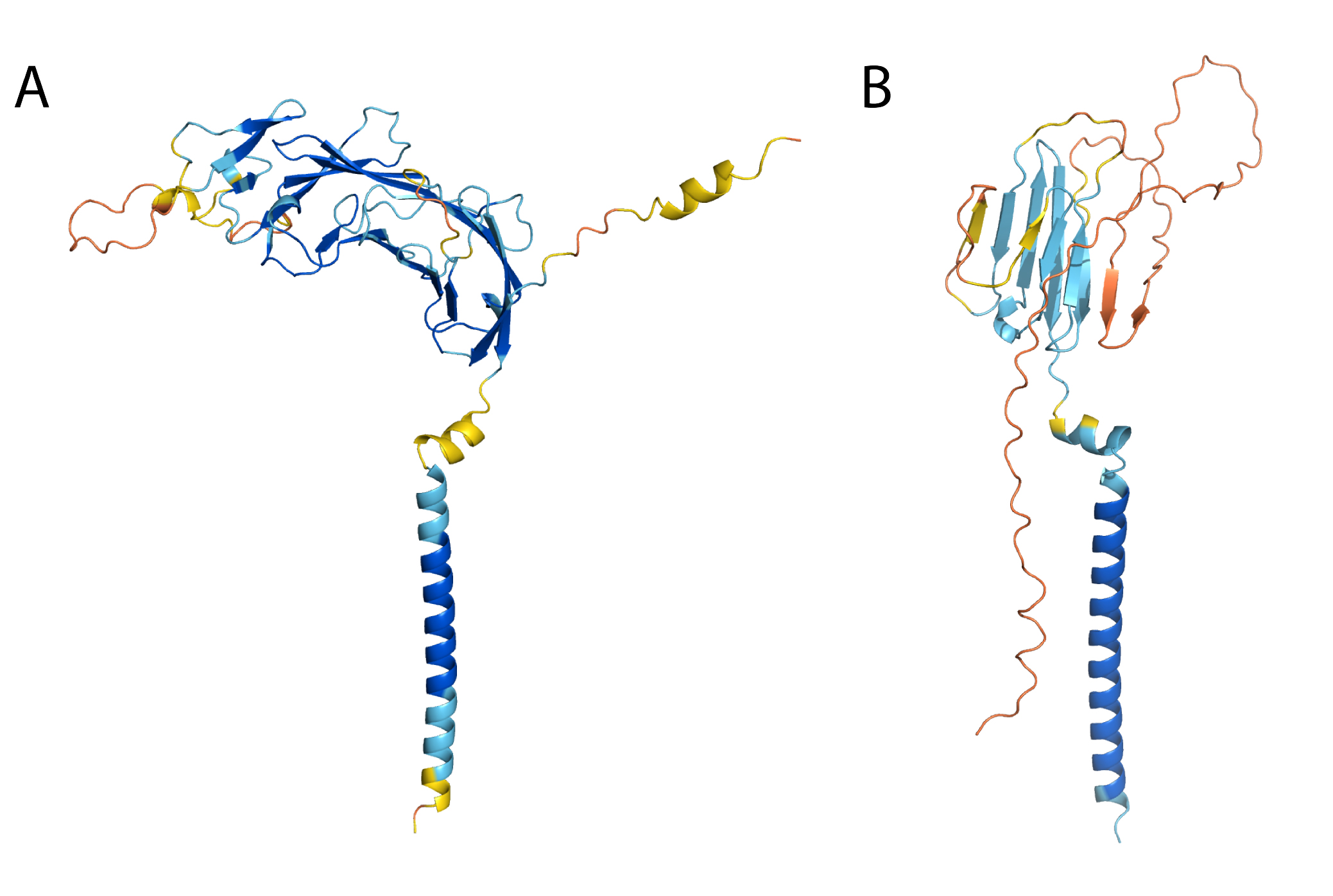

Supplement: Supplementary file 1 [file Image1.jpg]
